# RETRACTION: The Usefulness of Platelet‐Rich Plasma to Manage Skin Wounds: A Meta‐Analysis

**DOI:** 10.1111/iwj.70223

**Published:** 2025-02-11

## Abstract

**RETRACTION**: ChiL. , WangQ. , ZhongW. , PanX. , and LiY. , “The Usefulness of Platelet‐Rich Plasma to Manage Skin Wounds: A Meta‐Analysis,” International Wound Journal 20, no. 8 (2023): 3123–3130, 10.1111/iwj.14188.37128184 PMC10502258

The above article, published online on 01 May 2023, in Wiley Online Library (http://onlinelibrary.wiley.com/), has been retracted by agreement between the journal Editor in Chief, Professor Keith Harding; and John Wiley & Sons Ltd. Following an investigation by the publisher, all parties have concluded that this article was accepted solely on the basis of a compromised peer review process. The editors have therefore decided to retract the article. The authors did not respond to our notice regarding the retraction.

**RETRACTION**: L. Chi , Q. Wang , W. Zhong , X. Pan , and Y. Li , “The Usefulness of Platelet‐Rich Plasma to Manage Skin Wounds: A Meta‐Analysis,” International Wound Journal 20, no. 8 (2023): 3123–3130, 10.1111/iwj.14188.37128184 PMC10502258

The above article, published online on 01 May 2023, in Wiley Online Library (http://onlinelibrary.wiley.com/), has been retracted by agreement between the journal Editor in Chief, Professor Keith Harding; and John Wiley & Sons Ltd. Following an investigation by the publisher, all parties have concluded that this article was accepted solely on the basis of a compromised peer review process. The editors have therefore decided to retract the article. The authors did not respond to our notice regarding the retraction.

